# Population genetic variation characterization of the boreal tree *Acer ginnala* in Northern China

**DOI:** 10.1038/s41598-020-70444-w

**Published:** 2020-08-11

**Authors:** Hang Ye, Jiahui Wu, Zhi Wang, Huimin Hou, Yue Gao, Wei Han, Wenming Ru, Genlou Sun, Yiling Wang

**Affiliations:** 1 College of Life Science, Shanxi Normal University, Linfen, 041000 Shanxi China; 2 Changzhi University, Changzhi, 046011 Shanxi China; 3 Department of Biology, Saint Mary’s University, Halifax, NS B3H3C3 Canada

**Keywords:** Ecology, Evolution, Genetics, Plant sciences

## Abstract

Genetic diversity and differentiation are revealed particularly through spatio-temporal environmental heterogeneity. *Acer ginnala,* as a deciduous shrub/small tree, is a foundation species in many terrestrial ecosystems of Northern China. Owing to its increased use as an economic resource, this species has been in the vulnerability. Therefore, the elucidations of the genetic differentiation and influence of environmental factors on *A. ginnala* are very critical for its management and future utilization strategies. In this study, high genetic diversity and differentiation occurred in *A. ginnala*, which might be resulted from its pollination mechanism and species characteristics. Compared with the species level, relatively low genetic diversity was detected at the population level that might be the cause for its vulnerability. There was no significant relationship between genetic and geographical distances, while a significant correlation existed between genetic and environmental distances. Among nineteen climate variables, Annual Mean Temperature (bio1), Mean Diurnal Range (bio2), Isothermality (bio3), Temperature Seasonality (bio4), Precipitation of Wettest Month (bio13), Precipitation Seasonality (bio15), and Precipitation of Warmest Quarter (bio18) could explain the substantial levels of genetic variation (> 40%) in this species. The *A. ginnala* populations were isolated into multi-subpopulations by the heterogeneous climate conditions, which subsequently promoted the genetic divergence. Climatic heterogeneity played an important role in the pattern of genetic differentiation and population distribution of *A. ginnala* across a relatively wide range in Northern China. These would provide some clues for the conservation and management of this vulnerable species.

## Introduction

Genetic diversity and variation are manifested through phenotypic, chromosomal, and proteomic variations, which are revealed particularly through spatio-temporal environmental heterogeneity^1–3^. Geographical topology can play a key role in determining genetic divergence of species^4–6^. Climate conditions, in particular the most recent climatic oscillations, can also impact the evolution of the local organisms^7^. Under the changing conditions, if species unable to respond the environmental change, it will be at increased risk of extinction^8^. Indeed, species survival ultimately depends on its genetic diversity and variation^9^. Thus, investigating the genetic diversity and extent of genetic differentiation within/between populations, while estimating the impact of environmental factors and understanding the processes that maintain these variations not only are very useful toward gaining deeper insights into evolutionary history, but also are critical for the prudent formulation of conservation, management and utilization strategies^2,3,10–13^.

*Acer ginnala* (Aaceraceae) is a deciduous shrub/small tree that has monoecious and anemophilous pollination with an outcrossing breeding system^14–16^. It is a foundation species in many terrestrial ecosystems, distributing in Korea, Japan, Russia, Siberia, and China^15^. In China, this species primarily distributed in the northern regions, including Inner Mongolia, Hebei, Henan, Shanxi, and Anhui provinces, growing on cloudy slopes, gullies and valleys. *A. ginnala* has important economic value as the abundant gallic acid within leaves^17–21^ that can use as a functional material or novel herbal medicine with extracted galloyl derivatives^22^. However, the increased developments as an economic resource have accelerated the degradation of *A. ginnala* populations so that this species has been listed as a vulnerable plant in a recent nationwide biodiversity report^23^.

To provide theoretical basis for formulating effective and reasonable protection strategies, in our previous studies, the genetic diversity and differentiation of *A. ginnala* were firstly examined along an altitude gradient at Qiliyu in Shanxi, China^24,25^. In Qiliyu, high genetic diversity of *A. ginnala* was found. Significant differentiations in phenotype and genetics were observed in populations^24,25^. The level of genetic variation of the studied populations was observed to increase along an elevation gradient^26^, indicating the influence of climatic heterogeneity.

Although these studies revealed the level of genetic diversity in *A. ginnala*, a small number of individuals from a small-scale district were characterized; thus, the results provided very limited information on the genetic diversity and variations of this species. The extent and pattern of genetic differentiation of *A. ginnala* at relatively wide geographic scales remained unknown. Meanwhile, in northern regions of China, there is a complex geographical topology with more heterogeneous climate. How these conditions have affected the pattern of genetic variation across the present range of this species is also unclear.

Being a long-lived species, the geographical history events and climatic oscillations would be predicted to leave a signature on the level of genetic diversity and variation of *A. ginnala*. According to this hypothesis, significant genetic variation among populations/groups would provide an evidence and a significant relationship would be present between genetic differentiation and climatic-geographical factors.

In the last few years, microsatellites (SSR) have become one of the most popular codominant molecular markers used with applications in many different fields. High polymorphism and the relative ease of scoring represent the two major features that make microsatellites of large interest for many populations genetic studies^27–29^. Meanwhile, the sequence-related amplified polymorphism (SRAP) technology has been recognized as a useful molecular marker system for population genetics research^30–31^. It targets coding sequences and results in the identification of a number of codominant markers. SRAP is more reproducible than RAPD (Random Amplified Polymorphic DNA) and less complicated than AFLP (Amplified Fragment Length Polymorphism)^32^. Ferriol et al.^31^ reported that the information obtained from SRAP markers was more concordant with the morphological variations and the evolutionary history of the morphotypes than that found with AFLP markers.

**Figure 1:**
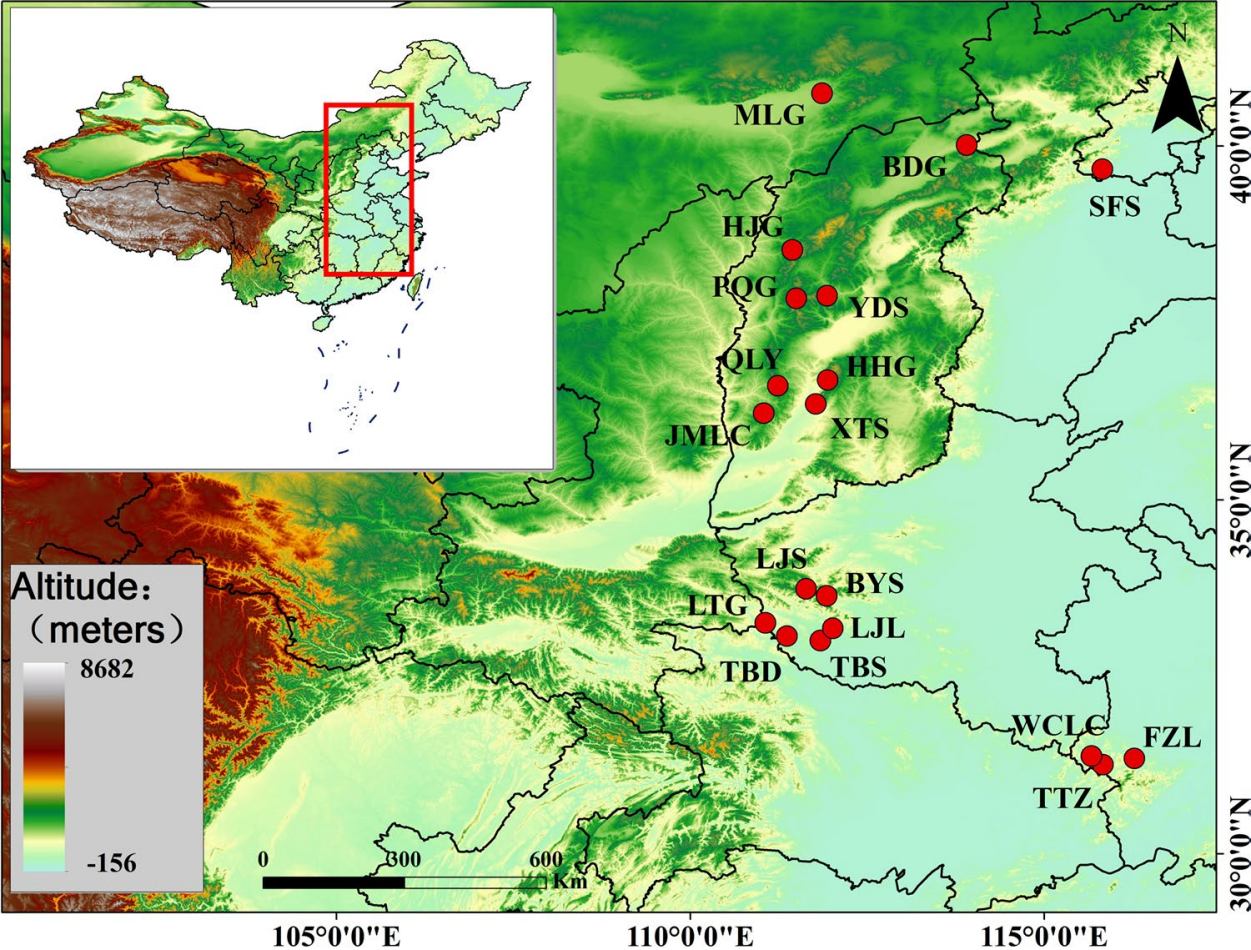
Sampled distribution sites of *Acer ginnala* populations in this study. The map was constructed using the ARCGIS 10.4 (https://desktop.arcgis.com/en/, ESRI). The Chinese administrative division data for mapping was downloaded from the National Geomatics Center of China (https://www.ngcc.cn/ngcc/, NGCC) with a scale of 1:400.

For this study, we presented the first investigation of the genetic variation of *A. ginnala* across a wide range in China using two types of molecular markers (SSR and SRAP), with a specific focus on genetic differentiation in the population, in order to understand whether geographical and climatic factors affected the genetic variation and genetic structure. In particular, the main objectives were to: estimate the genetic variability of *A. ginnala* populations and geographical groups; analyze the genetic structures and relationships of *A. ginnala* populations; verify the potential influences of spatial and environmental factors on population differentiation patterns. The combined analysis of molecular markers and eco-geographical data would provide beneficial knowledge for the utilization and conservation of this wild plant germplasm, for further revealing the evolutionary history of forest community in northern regions of China (Table 1, Fig. 1).

**Table 1:** Sampled populations of *Acer ginnala.*

Population	Latitude	Longitude	Altitude/m	Number of individuals (SRAP/SSR)
BDG, Badaogou, Shanxi	41°08′31.16″	114°08′16.09″	1,580	22/22
HJG, Haojiagou, Shanxi	38°32′11.08″	111°26′17.16″	1,450	16/16
HHG, Houhuigou, Shanxi	36°48′01.16″	111°45′05.51″	1,200	20/20
JMLC, Jiemiaolinchang, Shanxi	36°49′41.07″	111°56′59.42″	1,450	10/10
PQG, Pangquangou, Shanxi	37°52′17.67″	111°27′12.89″	1,800	21/21
QLY, Qiliyu, Shanxi	36°36′53.80″	111°14′03.80″	1,560	18/18
XTS, Xingtangsi, Shanxi	36°25′06.48″	111°46′27.15″	1,530	18/18
YDS, Yundingshan, Shanxi	37°53′18.38″	111°34′36.70″	1,000	10/10
SFS, Shangfangshan, Beijing	39°40′35.24″	115°49′17.37″	1,420	11/11
MLG, Meiligeng, Inner Mongolia	40°40′16.10″	109°26′29.39″	1,273	21/21
BYS, Baiyunshan, Henan	33°40′13.43″	111°49′50.34″	1,479	22/22
LJL, Laojieling, Henan	33°37′11.03″	111°43′44.91″	1,482	15/15
LJS, Laojunshan, Henan	33°44′47.46″	111°38′13.26″	952	10/10
LTG, Longtangou, Henan	33°31′01.88″	111°36′43.34″	1,560	17/17
TBD, Taibaiding, Henan	33°30′50.50″	111°36′40.67″	1,440	15/15
TBS, Tongbaishan, Henan	32°23′53.80″	113°09′37.80″	960	16/16
TTZ, Tiantanzai, Anhui	31°10′17.44″	115°46′04.85″	560	18/18
FZL, Foziling, Anhui	31°20′58.53″	116°16′32.11″	700	15/15
WCLC, Wochuanlinchang, Anhui	31°14′33.80″	115°50′13.80″	760	15/15

## Results

### Genetic diversity and variation of A. ginnala populations

For the SSR markers, a few loci were found to significantly deviate from HWE, three in the BDG population, four in the JMLC and XTS populations, five in the MLG and LJS populations, and one in the TTZ and WCLC populations. These loci were removed in the further analyses. There were 177 polymorphic loci in 179 putative genetic SSR loci, with the percentage of polymorphic bands (*PPB*) being 98.99%. A total of 170 bands were amplified with the SRAP marker, and 100% of the bands were polymorphic. Based on the SSR markers, the highest genetic diversity was present in the PQG population, followed by XTS and BYS (Table 2). According to the SRAP markers, the HHG population had the highest genetic diversity, followed by QLY and BYS (Table 2). At the species level, *A. ginnala* exhibited a high level of genetic diversity using the two types of markers (*I*_SSR_ = 0.561, *I*_SRAP_ = 0.5044; *H*e_SSR_ = 0.384, *H*e_SRAP_ = 0.3366), which was higher than the mean values at the population level (*I*_SSR_ = 0.086, *I*_SRAP_ = 0.057; *H*e_SSR_ = 0.056, *H*e_SRAP_ = 0.038) (Table 2). In addition, the mean inbreeding coefficients (*F*_IS_) was 0.4980 (by SRAP data) and 0.4972 (by SSR data) respectively.

**Table 2:** Genetic diversity of *Acer ginnala* populations.

Population	SSR			SRAP		
	*H*e	*I*	*PPB* (%)	*H*e	*I*	*PPB* (%)
BDG	0.038	0.062	17.32	0.035	0.053	11.18
HJG	0.044	0.068	15.64	0.028	0.045	11.76
HHG	0.059	0.091	19.55	0.070	0.106	21.76
JMLC	0.043	0.068	15.08	0.019	0.028	5.880
PQG	0.079	0.124	29.05	0.046	0.067	12.94
QLY	0.041	0.067	17.32	0.047	0.076	20.00
XTS	0.079	0.121	27.37	0.031	0.049	12.35
YDS	0.063	0.096	19.55	0.034	0.049	8.240
Group I	0.056	0.087	20.11	0.039	0.059	13.10
SFS	0.081	0.122	23.46	0.047	0.070	13.53
MLG	0.043	0.065	13.97	0.025	0.038	8.240
BYS	0.076	0.118	27.37	0.046	0.073	18.24
LJL	0.064	0.096	18.99	0.047	0.071	15.88
LJS	0.077	0.115	22.91	0.015	0.024	5.290
LTG	0.047	0.072	14.53	0.024	0.036	7.060
TBD	0.028	0.043	10.06	0.037	0.055	10.59
TBS	0.044	0.068	15.64	0.039	0.060	12.35
Group II	0.063	0.096	21.02	0.035	0.053	11.40
TTZ	0.060	0.091	18.99	0.031	0.050	12.94
FZL	0.057	0.086	17.88	0.054	0.082	17.06
WCLC	0.038	0.057	10.61	0.038	0.056	10.00
Group III	0.051	0.078	15.83	0.041	0.063	13.33
Mean	0.056	0.086	18.70	0.038	0.057	12.38
Species level	0.384	0.561	98.99	0.3366	0.5044	100.00

### Genetic structure and differentiation of A. ginnala populations

**Figure 2:**
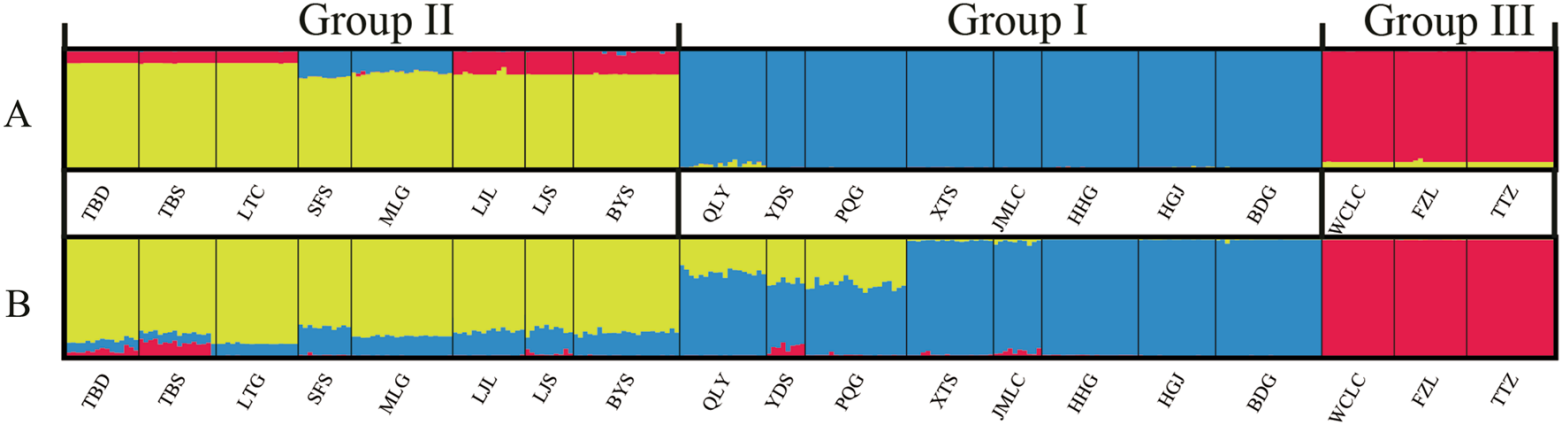
Genetic structure of *Acer ginnala* populations obtained by Bayesian analysis through Structure software for SRAP (**A**) and SSR (**B**), respectively.

**Figure 3:**
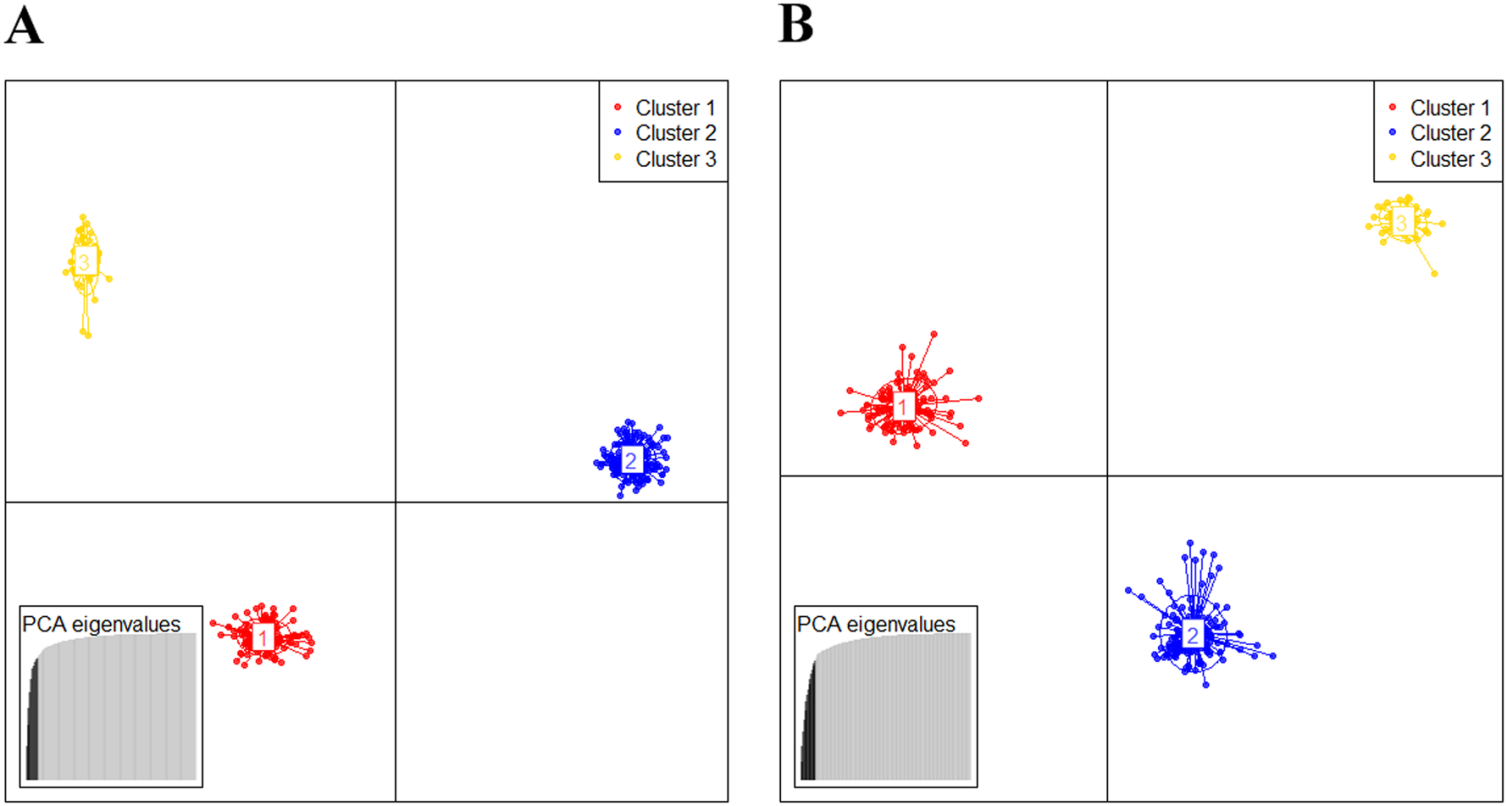
Genetic structure of the sampled populations as estimated by discriminant analysis of principal components (DAPC) based on SRAP (**A**) and SSR (**B**), respectively. (Cluster 1: Group I in STRUCTURE, Cluster 2: Group II in STRUCTURE, Cluster 3: Group III in STRUCTURE).

To reveal the patterns of genetic distribution of this species, we performed a population structure analysis using STURCTURE. The STRUCTURE results by both two markers suggested that the best grouping number (*K*) was 3 based on the Δ*K*, where all of the populations were divided into three groups (Fig. 2). Therein, eight populations, QLY, YDS, PQG, XTS, LMLC, HHG, HGJ and BDG were placed into Group I; TBD, TBS, LTG, SFS, MLG, LJL, LJS and BYS into Group II; the remaining three populations, WCLS, FZL and TTZ into the Group III. Some admixed individuals among populations were also observed, which indicated that the genetic lineage of some individuals comes from the mixture of different groups. In the DAPC analysis, which remained two discriminant function to distinguish ten principal components (PCs), three groups were present on the two main axes and a scatter plot of the discriminant analysis (Fig. 3). Every cluster was clearly differentiated using the two main DA eigenvalues, and were represented according the provinces of origin. The DAPC results were similar to the STRUCTURE results.

Based on the STURCTURE and DAPC results, we further analyzed the level of genetic diversity of the different groups with two types of markers. The results showed that Group I presented a relatively high genetic diversity, where the Shannon's Information index *I* was 0.087_SSR_ and 0.059_SRAP_, and the expected heterozygosity *H*e was 0.056_SSR_ and 0.039_SRAP_, respectively (Table 2), following by Group III and Group II (Table 2).

According to the SSR markers, the genetic differentiation between the populations was 84% (*Φ*_ST SSR_ = 0.84,* P* = 0.01), indicating that 16% of the total variance occurred within the populations. With SRAP markers, AMOVA (Analysis of Molecular Variance) revealed that 88% (*Φ*_ST SRAP_ = 0.88) of the total genetic variation was found among the populations (*P* = 0.01), whereas the remaining 12% of the total variation occurred within the populations (Table 3). The two types of markers indicated a high level of interpopulation genetic differentiation and low level of intrapopulation genetic differentiation in *A. ginnala* (Table 3). Among the different groups, the variation was ~ 40% (*Φ*_ST SSR_ = 0.40, *Φ*_ST SRAP_ = 0.42, *P* = 0.01) of the total variation, while there was ~ 60% variation within the groups (Table 3).

**Table 3:** Analysis of molecular variance (AMOVA) within/among *Acer ginnala* populations/groups.

Source	df	SS SSR/SRAP	MS SSR/SRAP	Est. Var SSR/SRAP	% SSR/SRAP	*Φ*_ST_ SSR/SRAP	*P* SSR/SRAP
Among populations	18	9,033.59/8,103.67	501.87/450.20	30.70/27.63	84/88	0.84/0.88	0.01
Within populations	289	1733.90/1,101.43	6.00/3.81	6.00/3.81	16/12		0.01
Total	307	10,767.50/9,205.10		31.45	100		
Among groups	2	2,222.34/3,106.76	1,111.17/1553.38	15.75/15.08	40/42	0.40/0.44	0.01
Within groups	305	8,545.16/6,098.34	28.02/20.00	23.63/20.99	60/58		0.01
Total	307	10,767.50/9,205.10		39.38/36.07			

The gene flow among populations was low, only ~ 0.040 (*N*_m SSR_ = 0.048, *N*_m SRAP_ = 0.034). Meanwhile, the gene exchange among groups was also low. Based on SRAP data, the migration occurred from Group I to II and the reverse was 0.1085 and 0.1124 respectively. Group II to III and the reverse was 0.1034, 0.1124, respectively. Group I to III and the reverse was 0.1109, 0.1101, respectively. For SSR data, the migration occurred from Group I to II and the reverse was 0.1158 and 0.1036, respectively. Group II to III and the reverse was 0.1132, 0.1136, respectively. Group I to III and the reverse was 0.1119, 0.1161, respectively (Table 4). The gene exchange either among populations or among groups was limited.

**Table 4:** The migration rate between groups of *Acer ginnala*.

	Group1 →	Group2 →	Group3 →
**SRAP**			
Group1	0.7806 (95% CI 0.62968–0.93152)	0.1124 (95% CI 0–0.26626)	0.1101 (95% CI 0–0.257492)
Group2	0.1085 (95% CI 0–0.253736)	0.7842 (95% CI 0.62823–0.94017)	0.1124 (95% CI 0–0.261556)
Group3	0.1109 (95% CI 0–0.256332)	0.1034 (95% CI 0–0.249224)	0.7776 (95% CI 0.62668–0.92852)
**SSR**			
Group1	0.7723 (95% CI 0.623536–0.921064)	0.1036 (95% CI 0–0.254324)	0.1161 (95% CI 0–0.267608)
Group2	0.1158 (95% CI 0–0.269072)	0.7832 (95% CI 0.628752–0.937648)	0.1136 (95% CI 0–0.264128)
Group3	0.1119 (95% CI 0–0.264584)	0.1132 (95% CI 0–0.26706)	0.7703 (95% CI 0.62526–0.91534)

### Influence of environmental heterogeneity on A. ginnala populations

Within the 19 bioclimative variables, seven variables were selected. They were bio1 (Annual Mean Temperature), bio2 (Mean Diurnal Range), bio3 (Isothermality), bio4 (Temperature Seasonality), bio13 (Precipitation of Wettest Month), bio15 (Precipitation Seasonality), and bio18 (Precipitation of Warmest Quarter). Among these seven bioclimatic variables, the first four (bio1, bio2, bio3, and bio4) were associated with the temperature dimension, while the last three (bio13, bio15, and bio18) were associated with the precipitation dimension. The PCA (Principal Component Analysis) showed that the explanatory percentage of the first two PCs (Principal Components) of the temperature dimension (45%) was estimated to be higher than that of the precipitation dimension (38%).

To assess whether geographic or environmental differences drove genetic divergence between populations, isolation-by-distance (IBD) and isolation-by-environment (IBE) tests were conducted using the Mantel test. The Spearman correlation showed no significant association between geographic and environmental distances (ρ_SRAP_ = − 0.1532, *P* = 0.284; ρ_SSR_ = − 0.0133, *P* = 0.474), or between genetic and geographic distances (ρ_SRAP_ = 0.1943, *P* = 0.051; ρ_SSR_ = 0.1549, *P* = 0.125). A significant relationship between genetic and environmental distances was found (ρ_SRAP_ = 0.4068, *P* = 0.001; ρ_SSR_ = 0.2647, *P* = 0.02). While, the partial Mantel test did not detect a significant correlation between genetic and geographic distance when conditioning on the environmental effect (ρ_SRAP_ = 0.2187, *P* = 0.307; ρ_SSR_ = 0.1643, *P* = 0.101). Nevertheless, there was a significant association between genetic and environmental distances, when the geographic distance was considered as a covariate in the partial Mantel test (ρ_SRAP_ = 0.2187, *P* = 0.037; ρ_SSR_ = 0.2701, *P* = 0.014). In terms of the joint effects of geographic and environmental distances in MMRR (Multiple Matrix Regression with Randomization) analysis, the geographic distance did not have a significant impact, while the environmental distance did impact the genetic distance significantly (r_SRAP_^2^ = 0.1686, β_geo_ = 0.4709, *P* = 0.052, β_env_ = 0.3.865, *P* = 0.001; r_SSR_^2^ = 0.1344, β_geo_ = 0.2847, *P* = 0.215, β_env_ = 0.1728, *P* = 0.003). These results revealed that the genetic variation of the populations was linearly correlated with climatic differentiation, but not geographical differentiation.

When conditioned on the geographic distribution, we found that over 40% of the variation (45.71% by SRAP data, 40.42% by SSR data) could be explained by climatic variables in dbRDA (distance-based redundancy analyses) (Table 5). The ANOVA further indicated that seven predictors (bio1, bio2, bio3, bio4, bio13, bio15, and bio18) significantly explained the genetic components of the population (*P* < 0.0001), and that bio2 and bio3 had the highest explanatory proportions for predicting the genetic variation of the population. Moreover, seven environmental variables could be separated into two categories: temperature and precipitation. Three bioclimatic variables (bio1, bio2, and bio3) of the temperature dimension had significant *F* statistics (adjusted R^2^ = 0.0259, 0.0400, and 0.0386, respectively, *P* < 0.05, Table 5) through SRAP data. According to the SSR data, two temperature variables (bio1 and bio3) had significant *F* statistics (adjusted R^2^ = 0.0067 and 0.0065, *P* < 0.05, Table 5). Only three variables (bio3, bio4, and bio13) were significantly correlated with the ordination axis1, while the other four variables (bio1, bio2, bio15, and bio18) were significantly correlated with axis 2 of dbRDA (Table 5).

**Table 5:** Summary of partial dbRDA, showing the significance of climatic variables (constrained factors) for explaining the variation in the genetic components.

	Var (SRAP/SSR)	Proportion (SRAP/SSR)	Adj R^2^ (SRAP/SSR)	*F* (SRAP/SSR)	P (SRAP/SSR)	Axis 1 (SRAP/SSR)	Axis 2 (SRAP/SSR)
Conditional	0.0122/0.0096	0.2055/0.1713					
Unconstrained	0.0321/0.0333	0.5429/0.5958					
Constrained	0.0271/0.0226	0.4571/0.4042					
bio1	0.0047/0.0035	0.0800/0.0619	0.0259/0.0067	1.6214/1.1431	0.006/0.017	− 0.0352/− 0.0230	− 0.0157/− 0.0003
bio2	0.0042/0.0032	0.0933/0.0637	0.0400/0.0087	1.4390/1.0573	0.034/0.151	− 0.9014/− 0.0723	0.2185/0.7319
bio3	0.0055/0.0035	0.0919/0.0616	0.0386/0.0065	1.8694/1.1417	0.001/0.013	0.4888/− 0.1502	0.0370/− 0.3001
bio4	0.0033/0.0031	0.0905/0.0599	0.0370/0.0046	1.1418/1.0135	0.210/0.382	0.0077/− 0.0002	− 0.0019/− 0.0034
bio13	0.0029/0.0031	0.0605/0.0588	0.0052/0.0034	0.9943/1.0311	0.438/0.264	0.0533/0.0777	− 0.0633/− 0.0473
bio15	0.0036/0.0031	0.0891/0.0587	0.0354/0.0034	1.2450/1.0643	0.109/0.116	− 0.0622/− 0.0296	0.0559/− 0.1175
bio18	0.0028/0.0333	0.0679/0.0599	0.0131/0.0047	0.9492/1.0120	0.530/0.420	− 0.0258/− 0.0296	0.0205/0.0238
Total	0.0592/0.0559	1.0000/1.0000					

## Discussion

*A. ginnala* contained a high genetic diversity at the species level in Northern China (Table 2). Genetic diversity is the culmination of the long-term evolution of species or populations^3,33,34^, which may be affected by multiple factors, such as the range of the species geographical distribution, genetic exchange, environmental conditions, and species characteristics^35–37^. As relates to geographical distribution, *A. ginnala* has a relatively wide distribution areas, from Southwest to Northeast China. Within a larger distribution area, species can generally possess higher genetic diversity^3,36,38^. Moreover, being a perennial tree, long lived *A. ginnala* could have more opportunities to accumulate mutations or specialized microstructures in different populations. In addition, *A. ginnala* is an insect-pollinated species^39,40^ with sexual propagation through seeds. This reproductive characteristic has significance for maintaining genetic diversity^36^. However, the genetic diversity at the population level was relatively low (Table 2), which might have been caused by two possible scenarios. Firstly, the inbreeding of plants can lower genetic diversity, which results from a reduction in population size, and leads to inbreeding depression^36,41^. Owing to human disturbance and destruction, *A. ginnala* population has being dwindled, becoming smaller than previously. Secondly, although *A. ginnala* is primarily insect-pollinated, self-pollination can occur, the *F*_IS_ of this species was ~ 0.5000 in our study.

There are reports that some plants have suffered from a general decline in pollinator insects, a phenomenon referred to a pollination crisis^42–44^. From the spring season, when *A. ginnala* flowers, an elevated rate of self-pollination might expect due to the presumable lack of effective pollinators^36^. Based on our field investigations, more adults and fewer seedlings were found in *A. ginnala* communities. Those populations might be exposed to greater genetic drift effects, resulting in low level of genetic diversity. Aside from anthropogenic factors, climate change might also affect this species populations. Low levels of genetic diversity might restrict the ability of a population to respond to changing environmental conditions^3,36,45^. Significantly environmental heterogeneity along the distribution areas of *A. ginnala* populations was suggested by the Mantel test and Spearman correlation between genetic and environmental distances, which was consistent with the previous reports^25,26^. Immigrant non-viability might have arisen from the local optimal for the environment; limiting the survival and reproduction of migrants^3,46,47^. On the other hand, most genetic variation (> 80%) was observed between populations, whereas ~ 10% of the differentiation existed within populations. High genetic variation values (Table 3) pointed to the presence of barriers to gene flow between populations, the gene flow was ~ 0.040 among populations (*N*_m SSR_ = 0.048, *N*_m SRAP_ = 0.034) in this study.

The seeds of *A. ginnala* have a large wing and can be dispersed by the wind, the potential for exchange might be more sensitive to geographic barriers than that of other wind-pollinated and wind-dispersed species. In the *A. ginnala* distribution areas, the discontinuous distribution of mountains, such as Taihang Mountains and Wuling Mountains, provide a complex landform that likely blocks gene exchange among populations^48–50^. These factors presumably isolated populations; thus, promoting population differentiation by limiting the potential for gene exchange. Interestingly, the Mantel test and Spearman correlation revealed no significant relationship between genetic and geographical distances. For *A. ginnala*, gene flow might also be obstructed by the pollination pattern. Being an insect pollinated species, pollen-mediated gene flow was generally limited, as the range of most pollinators might be less than 20 km^36^, and they tend to visit neighboring plants^51^. Thus, at a relative larger-scale area, *A. ginnala* populations might be geographically and/or ecologically too isolated to be connected via pollen exchange. Besides, the considerable genetic differences between populations might also be attributed to the absence of generative reproduction^52,53^. During the field observations, we found that some populations ruled out generative reproduction, due to the absence of flowering individuals, or their inability to produce fruit.

STURCTURE and DPAC analyses divided all of the studied populations into three clusters (Figs. 2, and 3). Group I and III contained relative higher genetic diversity among all examined populations (Table 2). Populations of Group I was located in the North Qinling Mountains and West Taihang Mountains, while the Group III populations were located in the South Qinling Mountains. The physiographic heterogeneity and topographical diversity of these regions lead to relatively variable and unstable climatic conditions^54^, which might favor higher genetic variation within Group I and III. Meanwhile, observed genetic differences between three groups were relatively large and statistically significant. Climate heterogeneity and/or discontinuous mountains in Northern China could segment large populations into multiple-small fragmented populations, further block the gene exchange among groups (*N*_m_ < 1 according to both two markers, Table 4), and then enhance differentiation between groups^49^.

The genetic divergence of populations was suggested to be correlated with both geographic distance and environmental heterogeneity^47^. In the present study, geographic distance was not correlated with environmental distance. When considering the combined effects of environmental and geographic distances, environmental distance was found to affect genetic distance significantly. These results suggested that IBE (Isolation by Environment) might have played an important role in shaping the genetic divergence of populations, corresponding to the environmental heterogeneity that occurred for *A. ginnala*. As revealed by dbRDA analysis, temperature and precipitation are frequently found to play prominent roles as a driver for genetic variation in various plant species^47,55–61^. Furthermore, in our study, the temperature seems to be more overwhelming and dominate to affect the genetic differentiation of *A. ginnala* than the precipitation variables. Within the studied populations, each was located in areas under different environment conditions. The maximum of Mean diurnal range (bio2) was 116 in the MLG population, and the minimum was 78 in the FZL population. For Isothermality (bio3), it was from 270 in the BYS population to 236 in the BDG population. Meanwhile monsoon rainfall may occur due to the orientation of different mountains, such as Qinling and Taihang Mountains, in the geographical distribution of *A. ginnala*^62–66^. Bio15 had the highest adjusted *R*^2^ value between three major precipitation ranges (Table 5), which is Precipitation seasonality. Therefore, the populations could demographically be isolated by environmental climate heterogeneity that might promote genetic divergence within *A. ginnala*.

In summary, the higher genetic diversity occurred in the *A. ginnala* species level, while the relatively lower genetic diversity on the population level. Significant genetic variation and differentiation was found to happen among populations or groups. The heterogeneous ecological environment affected and shaped the spatio-temporal genetic pattern of *A. ginnala* in Northern China. These results would provide some clues for the conservation, management and utilization of this vulnerable species. However, it should be noted that rather than individual environmental variables acting independently to shape the distribution patterns of the genetic variation of species, it was most likely that the interdependencies of environmental variables exerted direct and indirect effects on genetic divergence within and between species.

## Methods

### Sample collection

Nineteen *A. ginnala* populations were collected based on their geographical distribution (Table 1, Fig. 1), which almost covered its entire distribution across Northern China. Among these populations, 10–30 individuals were sampled from each population, with a minimum distance of 30 m between any two individuals. A total of 310 individuals were sampled. Healthy and young leaves were collected and immediately preserved with silica gel for DNA extraction, with the sampled populations being primarily from five provinces or regions. Eight *A. ginnala* populations were located in Shanxi Province, six in Henan Province, three in Anhui Province, one in Beijing City, and one in Inner Magnolia. The longitude, latitude, and altitude of each sample site were quantified using a global position system (Table 1).

### Primer selection and PCR amplification

Genomic DNA from the sampled individuals was extracted using the modified CTAB method^67^. The quality of the DNA was determined using an ultraviolet spectrophotometer and the electrophoresis on 0.8% agarose gel^68^. Following extraction, the DNA was stored at -20 ℃ for further use.

SSR markers were obtained from primers previously developed for *Acer mono*^49,69^ and *Acer capillipes*^70^. Subsequently, these primer pairs were selected to amplify 19 random DNA samples (one for each population) for the pre-experiments (primer screening). These abided by the following principles: the primer length was controlled between 18 and 25 bp, the GC content was from 40 to 60%, and the annealing temperature was from 50 to 60 °C. Hairpin structures, dimers, hat structures, and mismatches were avoided as much as possible. The primers were synthesized by Shanghai Sangon Biological Engineering Technology (Shanghai, China). Finally, ten pairs of polymorphic SSR primers with distinct bands and high stability were selected to amplify 310 individuals of the *A. ginnala* populations (Supplementary Table S1). The 20 μL reaction contained 20 ng/L DNA templates, 2 μL Mg^2+^, 2.5 μL dNTPs, 2 μL each primer, 1μL *Taq* DNA polymerase, and 7.5 μL double-distilled water. The reaction procedure included 95 °C modification (5 min), 94 °C denaturation (30 s), 55 (56, 57, 58) °C annealing (45 s), 72 °C extension (65 s), 36 cycles, and a final extension for 9 min at 72 °C.

While for SRAP markers, according to the primer design principles developed by Li and Quiros’s^30^, we also referred to the combination mode of forward and reverse primers in the closely related species of the genus *Acer* designed by Liu^71^. After primer screening, ten pairs of primers with clear and stable bands were also selected (Supplementary Table S1) to amplify all sampled individuals. The 20 μL PCR reaction system contained 50 ng/L of DNA templates, 2 μL 10 × PCR buffer, 2.5 μL dNTPs (0.5 μmol/L), 2 μL each primer (0.2 μmol/L), 1μL *Taq* DNA polymerase (0.08 U/μL; Takara, Shiga, Japan), and 10.5 μL double-distilled water. The reaction procedure included initial denaturation at 94 °C (5 min), followed by 36 cycles at 94 °C (40 s), annealing at Tm temperature under different primers (25 s), extension at 72 °C (65 s), and final extension at 72 °C (10 min).

The PCR amplified products were separated by polyacrylamide gel electrophoresis (12%), with an electrophoresis time of ~ 2.5 h at 200 V, and the fragments were visualized by silver staining.

### Genetic diversity and differentiation estimation

For both two molecular data, the genetic diversity parameters, such as the percentage of polymorphic bands (*PPB*), Shannon's Information index (*I*) and expected heterozygosity (*H*e) were calculated using GENALEX^72^. Additionally, departures from Hardy–Weinberg equilibrium (HWE) were tested also using GENALEX^72^ for SSR data. To elucidate the extent of genetic variation between and within populations or groups, the hierarchical analysis of molecular variance (AMOVA) was performed in GENALEX^72^. The groups were assigned according to the STRUCTRE results, DAPC results and geographical distribution of the studied populations. The gene flow was calculated by using the formula: *N*_m_ = (1 − *F*_ST_) /4*F*_ST_ to estimate the migration rates among populations. To further investigate gene flow among the three groups, BAYESASS software^73^ was used. The burn‐in period of the model was set at 1 × 10^6^, while MCMC iterations were set at 1 × 10^7^. Several instances of the model were run with different starting seeds. Results were similar among runs, and only the values from the first run were reported. The inbreeding coefficients (*F*_IS_) of this species was also estimated using BAYESASS^73^.

### Population structure investigation

STRUCTURE analysis^74^, underlying the model of Bayesian methods, was often used to delineate the clusters of genetically similar individuals, to reveal the patterns of genetic distribution of the species. A non-admixture model was applied to STRUCTURE with a priori sample localities. The posterior probability of grouping number (*K* = 1–19) was estimated by 15 independent runs using two-million steps Markov chain Monte Carlo (MCMC) replicates after one-million-steps burn-in for each run to evaluate consistency. The optimum *K* value was determined using the Delta *K* method (Δ*K* statistic) in STRUCTURE HARVESTER^75^. These 15 runs were aligned and summarized using CLUMPP.

To further confirm the population genetic structure, a discriminant analysis of principal components (DAPC) was conducted using the R package ‘adegenet’^76^. The genetic data was initially transformed according to Principal Component Analysis (PCA). These components explained most of the genetic variation based on PCA was then used to perform linear Discriminant Analysis (DA), which provided variables to describe the genetic groups that minimized the genetic variance within populations, while maximized the variation between populations.

### Environmental heterogeneity influence

To investigate the influence of the environment on *A. ginnala* variation, we extracted the environmental factors with DIVA-GIS software^77^ and determined the key environmental factors by the “vif” function in the R package ‘usdm’^78^. Variables with a high variance inflation factor (VIF). were removed to reduce multicollinearity. The VIF values of all the remaining variables were < 10, where finally seven bioclimatic variables were retained for further analyses.

To test how the geographic distance and environmental differences affect the genetic composition of *A. ginnala*, the Mantel test of Spearman correlation was performed among genetic, geographic, and environmental (climatic) distances using R package ‘vegan’^79,80^. Pairwise *F*_ST_ was calculated between populations based on Nei’s^81^ method, then *F*_ST_/(1 − *F*_ST_) was used to estimate the genetic distance metric. Geographic distances were estimated using Euclidean distances, according to three dimensional factors (latitudes, longitudes, and altitudes). Environmental distances with seven bioclimatic variables were also calculated using Euclidean distances. A partial Mantel test between genetic and environmental distances controlled for the geographic distance was also performed. Further, multiple matrix regression with randomization (MMRR)^82^ was performed using R package ‘PopGenReport’^83^ to test whether genetic distances responded to variations in geographic and/or environmental distances. The joint effects of both geographic and environmental distances on the genetic distances were also examined. Regression coefficients of the Mantel test (ρ) and MMRR (β), and their significance, were estimated based on 9,999 random permutations. Additionally, partial distance-based redundancy analyses (partial dbRDA) were performed to explain the effects of climatic variables on the genetic distribution of populations using R package ‘vegan’^79,80^. The generalize linear regression model (GLRM) was employed to test the effects of climatic variables on populations.

## Supplementary information

Supplementary Information.

## Data Availability

The datasets supporting the conclusions of this article are included within the article and its additional files. The datasets used and/or analyzed during the current study are available from the authors on reasonable request (Hang Ye, SXSDyehang@hotmail.com; Yi-ling Wang, ylwangbj@hotmail.com).
