# Supplementary material for: Population genetic variation characterization of the boreal tree Acer ginnala in Northern China
Source: Sci Rep. 2020 Aug 11;10:13515. doi: 10.1038/s41598-020-70444-w (PMC7419535; doi:10.1038/s41598-020-70444-w)
Supplement: Supplementary file 1 — Supplementary Information. [file 41598_2020_70444_MOESM1_ESM.docx]

Table S1. The forward and reverse Simple Sequence Repeats (SSR) and sequence related amplified polymorphism (SRAP) primers information for this study

| SSR | | | | |  | SRAP | | | |
| --- | --- | --- | --- | --- | --- | --- | --- | --- | --- |
| Number | Primer name | Primer sequences (5′–3′) | *T*a °C | Repeat motif |  | Number | Primer pairs | Primer sequences (5′–3′) | *T*a °C |
| 1 | Am116 | F: AACGCTACCGACTTCGCCAACT R: TGGAGGTCAAGTGCTGGAAACAA | 58 °C | (CT)_20_ |  | 1 | me2/em1 | me2: TGAGTCCAAACCGGAGC em1: GACTGCGTACGAATTAAT | 51 °C |
| 2 | Am118 | F: GAGGGAGGAGGCTGAGAAGA R: TATCAAAGAAGCCAAGGAAGGTG | 58 °C | (CT)_16_ |  | 2 | me2/em6 | me2: TGAGTCCAAACCGGAGC em6: GACTGCGTACGAATTGCA | 55 °C |
| 3 | Am258 | F: CCGGTGCATCTATCTCCAT R: CATCCATAAAGTAAAAATTGAGGG | 58 °C | (CT)_17_ |  | 3 | me2/em9 | me2: TGAGTCCAAACCGGAGC em9: GACTGCGTACGAATTGAT | 52 °C |
| 4 | Am340 | F: CGGAGCCAACTTGAGAGTAGAG R: ATTGAAGGTCCTTAATCCACGTC | 58 °C | (AG)_22_ |  | 4 | me4/em1 | me4: TGAGTCCAAACCGGAGC em1: GACTGCGTACGAATTGAT | 53 °C |
| 5 | Am607 | F: CACACATGGGCTTCTCTATGAGT R: CATCCGCCAGTTGGTGAAT | 58 °C | (AG)_15_ |  | 5 | me4/em6 | me4: TGAGTCCAAACCGGAGC em6: GACTGCGTACGAATTGCA | 50 °C |
| 6 | Am742 | F: AGAACAGGCGGAGAGTTTCGAGTC R: CCCGACGACAACCACCCAT | 58 °C | (AG)_17_ |  | 6 | me6/em1 | me6: TGAGTCCTTTCCGGTAA em1: GACTGCGTACGAATTAAT | 51 °C |
| 7 | Aca6 | F: CATGCATGCTCATGCACAAGA R: TAGCATCTACCGGATACATTG | 55°C | (AC)_16_ |  | 7 | me8/em1 | me8: TGAGTCCAAACCGGTGC em1: GACTGCGTACGAATTAAT | 57 °C |
| 8 | Aca17 | F: TCAAAACAGTTACCAAAAACC R: TCAGAAATTCACAGGCCAACT | 55°C | (TC)_17_(AC)_13_ |  | 8 | me9/em6 | me9: TGAGTCCAAACCGGTAG em6: GACTGCGTACGAATTGCA | 56 °C |
| 9 | Aca22 | F: TATCATCAAACATTGCTACTC R: ATTCGGTGTCGTGTTCGTATT | 56°C | (AT)_2_(AC)_15_ |  | 9 | me9/em9 | me9: TGAGTCCAAACCGGTAG em9: GACTGCGTACGAATTGAT | 52 °C |
| 10 | Aca24 | F: TTCAACCACTCAATTTCAACA R: AAGGTCAAATCTTGATCTATC | 57°C | (AG)_5_(AC)_6_ |  | 10 | me9/em10 | me9: TGAGTCCAAACCGGTAG em10: GACTGCGTACGAATTCAG | 55 °C |

F: forward primers. R: reverse primers. *T*a: annealing temperature.
